# Evaluating teacher grading during the pandemic: An analysis of how teacher grades in 2020 compared to exam grades during ‘normal times’

**DOI:** 10.23889/ijpds.v8i2.2303

**Published:** 2023-09-14

**Authors:** Tim Stratton, Chifumnanya Okpala

**Affiliations:** 1 Ofqual, Coventry, United Kingdom

The aim was to evaluate teacher grades awarded in 2020 after GCSE and A-level exams were cancelled due to the pandemic. We identified if there were changes in the relationships between student, school and subject-level features and grades awarded by teacher judgement in 2020 when compared to pre-pandemic years.

A multi-level modelling analysis was conducted using the GRADE dataset which links data on pupil characteristics with results data and university application data. The model outputs identifying the relationship between student, school and subject characteristics and GCSE and A-level results were compared between 2020 and normal exam years to identify any notable changes.

A further study focussed on whether university application patterns had an influence on teacher judgements at A-level, since teachers may have known students’ application decisions when deciding on grades. For this analysis university tariff and the degree subject applied to by each student were key variables.

Analysis showed that across both GCSEs and A-levels there was an average increase in results by around half a grade in 2020. There was also less variation in grades in 2020. However, the majority of relationships between grades and other features studied had not substantially changed.

Prior attainment was by far the strongest predictor of grades, but this relationship was slightly stronger in 2020 compared to previous years.

Subjects with more non-exam assessment tended to be those with the largest increase in grades, as well as ‘facilitating’ subjects at A level. There was also a decrease in the attainment gap between male and female students at A level.

The follow up study indicated that teachers were not influenced by student’s application decisions when allocating grades.

Findings suggest that teacher grades did not introduce any substantial sources of bias or different patterns of grading in 2020. This research helped to inform policy decisions around national assessment in England in 2021, as well as contributing to the broader understanding of direct grading by teachers.

